# Screening for neuropsychological impairment in COPD patients undergoing rehabilitation

**DOI:** 10.1371/journal.pone.0199736

**Published:** 2018-08-01

**Authors:** Antonia Pierobon, Laura Ranzini, Valeria Torlaschi, Elisa Sini Bottelli, Anna Giardini, Claudio Bruschi, Roberto Maestri, Simona Callegari, Rita Raccanelli, Marinella Sommaruga

**Affiliations:** 1 Psychology Unit, Istituti Clinici Scientifici Maugeri Spa SB, IRCCS, Montescano (PV), Italy; 2 Department of Pulmonary Rehabilitation, Istituti Clinici Scientifici Maugeri Spa SB, IRCCS, Montescano (PV), Italy; 3 Department of Biomedical Engineering, Istituti Clinici Scientifici Maugeri Spa SB, IRCCS, Montescano (PV), Italy; 4 Cardiorespiratory Rehabilitation Unit, Istituti Clinici Scientifici Maugeri Spa SB, IRCCS, Camaldoli (MI), Italy; 5 Clinical Psychology and Social Support Unit, Istituti Clinici Scientifici Maugeri Spa SB, IRCCS, Camaldoli (MI), Italy; Chiba Daigaku, JAPAN

## Abstract

**Introduction:**

Chronic obstructive pulmonary disease (COPD) is a complex multi-component disorder characterized by progressive irreversible respiratory symptoms and extrapulmonary comorbidities, including anxiety-depression and mild cognitive impairment (MCI). However, the prevalence of these impairments is still uncertain, due to non-optimal screening methods. This observational cross-sectional multicentre study aimed to evaluate the prevalence of anxiety-depressive symptoms and MCI in COPD patients, identify the most appropriate cognitive tests to screen MCI, and investigate specific cognitive deficits in these patients and possible predictive factors.

**Materials and methods:**

Sixty-five stable COPD inpatients (n = 65, aged 69.9±7.6 years, mainly stage III–IV GOLD) underwent the following assessments: Hospital Anxiety and Depression Scale (HADS), Geriatric Depression Scale (GDS) or Beck Depression Inventory-II (BDI-II), Mini-Mental State Examination (MMSE), Montreal Cognitive Assessment (MoCA) and a complete neuropsychological battery (ENB-2) including different cognitive domains (attention, memory, executive functions, and perceptive and praxis abilities).

**Results:**

Moderate-severe anxiety was present in 18.5% of patients and depressive symptoms in 30.7%. The prevalence of MCI varied according to the test: 6.2% (MMSE), 18.5% (MoCA) and 50.8% (ENB-2). In ENB-2, patients performed significantly worse compared to Italian normative data on digit span (5.11±0.9 vs. 5.52±1.0, p = 0.0004), trail making test-B (TMT-B) (176.31±99.5 vs. 135.93±58.0, p = 0.004), overlapping pictures (26.03±8.9 vs. 28.75±8.2, p = 0.018) and copy drawing (1.370.6 vs. 1.61±0.5, p = 0.002). At logistic regression analysis, only COPD severity (p = 0.012, odds ratio, OR, 4.4 [95% CI: 1.4–14.0]) and anxiety symptoms (p = 0.026, OR 4.6 [1.2–17.7]) were significant and independent predictors of the deficit in copy drawing, which assesses visuospatial and praxis skills.

**Conclusion:**

Given the prevalence of neuropsychological impairments in COPD patients, the routine adoption in rehabilitation of screening tools for mood and cognitive function, including digit span, TMT-B and copy drawing, may be useful to detect psychosocial comorbidities and personalize the rehabilitative program.

## Introduction

Chronic obstructive pulmonary disease (COPD) is a complex multi-component disorder characterized not only by progressive and largely irreversible airflow limitation, shortness of breath, cough, and expectoration, but also by extrapulmonary effects [1,2]. The brain, in particular, may be vulnerable to the systemic effects of COPD. Several features of the disease could contribute to impair cognitive functions, including hypoxemia and comorbid cardiovascular disease. However, the pattern of cognitive dysfunction in COPD is different from that in multi-infarct dementia, and memory has been shown to be worse in individuals with chronic cerebrovascular disease than in those with COPD [3].

Studies on cognitive performance in patients with COPD compared to healthy subjects found a poorer performance mostly on cognitive tests assessing attention, memory and executive functions; nevertheless, the prevalence of cognitive deficits due to mild cognitive impairment (MCI) is controversial [4]. MCI is a syndrome characterized by a significant cognitive decline, greater than that expected for age and education level and with no impact on the activities of daily life [5,6]. MCI prognosis is still unclear: some patients remain in a stable condition, others improve and return to normal. There are different subtypes of MCI: single domain amnestic MCI, multiple domain amnestic MCI, single domain non-amnestic MCI, and multiple domain non-amnestic MCI [7,8]. Villeneuve et al. found MCI in 36% of patients with COPD, where the prevalent type was the single domain non-amnestic type, with predominant deficits in attention and executive functions. In the last few years, several studies have assessed the prevalence of cognitive deficits in COPD, but using different modes to assess MCI: with a consensus panel according to standardized criteria or using a complete neuropsychological battery or with a single screening test [4, 9, 10].

In clinical practice, it is desirable to have sound tools to screen for MCI, but an extensive neuropsychological assessment is time-consuming and requires specific expertise. The ideal instrument for MCI screening should be short, easy to administer and to correct, accepted by patients, independent of language or education constraints, psychometrically sound, and it should assess the as many cognitive domains as possible [11]. While the Mini-Mental State Examination (MMSE) is the most commonly used test for screening cognitive impairment (3), also the Montreal Cognitive Assessment (MoCA) has been demonstrated to be valid for detecting MCI in patients with COPD [4, 12, 13]. The MoCA is a cognitive screening instrument that was developed to detect MCI, and it seems particularly useful for identifying non-amnestic MCI [14, 15]. Nevertheless, the best way to detect MCI would be, if possible, through a complete battery, which can provide accurate information about the presence of specific cognitive deficits. The Esame Neuropsicologico Breve 2 (ENB-2) is a complete neuropsychological battery that can detect the different types of MCI due to the wide range of cognitive domains included: attention, memory, executive functions, and perceptive and praxis abilities [16, 17].

It is also well known that COPD patients have a higher prevalence of depressive symptoms than healthy subjects (24.6% [95% confidence interval, CI: 20.0–28.6] vs. 11.7% [9–15.1]) [18], though it seems that depression may account for only 1–2% of the variance in cognition in COPD [3]. In addition, anxiety characterizes the life of COPD patients and could have an important effect on COPD health outcomes [19, 20]. Moreover, there is evidence that anxiety is associated with non-anamnestic MCI, which involves executive dysfunctions in COPD patients [21, 22], but the specific association between anxiety and MCI is still not fully clear [23]. Recent studies have reported a significant association between diseases severity, depressive symptoms, anxiety, MCI, self-reported adherence and sleep quality in COPD patients, but only questionnaires (MoCA or MMSE) were used to detect MCI [13, 24].

Hence, a comprehensive psychological and neuropsychological assessment remains the most effective way to assess MCI and its associated variables, such as anxiety and depression. The aims of the present observational cross-sectional multicentre study were: 1) to evaluate the prevalence of anxiety and depressive symptoms and MCI in COPD patients; 2) to identify the most appropriate cognitive tests for screening MCI; 3) to detect possible specific cognitive deficits in these patients; and 4) to investigate which factors might predict specific neuropsychological impairments.

## Materials and methods

### Subjects

All patients with COPD consecutively admitted to the Clinical Scientific Institutes Maugeri IRCCS Departments of Pulmonary Rehabilitation of Montescano (PV) and Camaldoli (MI) for inpatient rehabilitation between September 2013 and March 2014 were eligible if diagnosed as stage II–IV according to Global initiative for Chronic Obstructive Lung Disease (GOLD) criteria and if in a clinically stable condition (no exacerbations in the last 3 months) with optimized stable pharmacological therapy (inhalation therapy with long-acting anticholinergic and/or β2-agonists, inhaled corticosteroids when needed). Exclusion criteria were: severe medical conditions that did not enable evaluation (severe chronic inflammatory diseases, chronic heart failure, neoplastic diseases, acute respiratory diseases), no Italian education or relapse into illiteracy, severe visual-perceptive deficits, low subjective motivation or unwillingness to perform the evaluation, severe psychiatric disorders (by medical psychiatric evaluation) and severe cognitive deterioration (MMSE≤18.3) [25]. The COPD patients included in this study were a subset of patients described elsewhere [13] who had undergone a broader neuropsychological investigation. All patients gave written informed consent to participate in the study. This work is part of a broader research project, approved by our institutional Review Board and Central Ethical Committee (CEC) of the Istituti Clinici Scientifici Maugeri SpA SB (approval number: CEC N.927, 27/06/2013).

### Procedure

All patients underwent a comprehensive rehabilitation program as part of routine clinical practice consisting of: educational sessions, physical exercise training (cycloergometer and/or treadmill, arm ergometer), respiration against resistance, calisthenics, psychological counselling, and metabolic evaluation with a personalized diet when needed. During the first week of admission, all patients underwent an individual psychological and neuropsychological assessment by means of: the Hospital Anxiety and Depression Scale (HADS) [26, 27], 30-point Geriatric Depression Scale (GDS) [28], Beck Depression Inventory (BDI-II) [29, 30], MMSE [31, 25], MoCA [14, 15], and a broad validated neuropsychological battery for the Italian population, the Esame Neuropsicologico Breve-2 (ENB-2) [16]. The psychological and neuropsychological assessment was divided into two sessions: 1) MMSE and psychological test, and 2) MoCA and ENB-2 (administered a few days later in order to avoid an interference effect). Trained psychologists evaluated the patients according to standardized administration and scoring procedures. The patients were supported throughout the testing period to maintain their motivation and elicit an optimal level of performance; a break was always allowed if necessary.

### Instruments

The MMSE consists of a brief 30-point questionnaire, and was used to exclude patients with severe deterioration (<18.3). Scores were adjusted for age and education according to the Measso et al. distribution [25]. Scores in the range of 18.3–23.8 could indicate moderate (not severe) cognitive impairment, with a high probability that they reflected a diminished performance for reasons other than aging or poor education. Therefore, in this study we defined MCI as present when MMSE score was 18.3–23.8 and not present when MMSE scores were >23.8.

The HADS was developed to identify anxiety and depressive symptoms among patients in non-psychiatric hospital clinics. In this study, we used the Anxiety subscale only (HADS-A). The Depression subscale (HADS-D) was not considered since we used the GDS to detect depressive symptoms in patients aged ≥65 years and the BDI-II for patients aged <65 years. The BDI-II scores were adjusted for sex and age and divided into percentile points. It is possible to consider jointly the GDS and the BDI-II scores, since both of them divide the depressive symptoms into four comparable categories: no, mild, moderate and severe.

The MoCA is a cognitive screening instrument developed to detect MCI. It is a simple 10-min paper-and-pencil test and with a maximum score of 30. It assesses multiple cognitive domains including memory, language, executive functions, visuospatial skills, calculation, abstraction, attention, concentration, and orientation. The MoCA is freely accessible for clinical and educational purposes, and is validated in 36 languages including Italian (www.mocatest.org). Raw neuropsychological scores were adjusted for age, education and sex, which were entered into a multiple linear regression analysis to partial out their possible overlapping effect. The results of the multiple regression analyses were entered into a regression equation to calculate a correction factor for each subject. Adjusted scores were obtained by adding or subtracting the contribution of concomitant variables from the original scores. The cut-off value was defined as the score at or below which the probability that an individual belongs to the normal population was less than 0.05. The adjusted scores were classified into five equivalent scores (ES) endowed with an ordinal relationship: 0 = scores lower than the outer 5% tolerance limits; 4 = scores higher than the median value of the sample; 1, 2 and 3 were obtained by dividing into three equal parts the area of distribution between 0 and 4. Hence, ES = 0 (score interval: 0–17.362) was considered always as below the norm, ES = 1 (score interval: 17.363–19.500) was borderline and here could be considered not normal according to the clinical condition, settings and clinical judgement. Therefore, in this study we defined MCI as present for ES = 0–1 and not present for ES = 2–4 [15].

The ENB-2 battery contains specific tests already available in the literature and readjusted in the Italian population for age and education [16]; for each ENB-2 test, two values are available: mean ± standard deviation (SD) and a global normative value, obtained by a weighted average of normative scores in the 7 levels of age-education. In each neuropsychological test, the impaired score represents a value equal to or worse than 5% of the normative sample. The tests of ENB-2 are grouped into three cognitive domains, according to the division presented by Mapelli et al.: memory, attention/executive function and visuospatial and praxis skills. The cognitive domain of attention includes trail making test A (TMT-A) and B (TMT-B); the domain of memory includes digit span, the Babcock story recall test (BSRT) and interference memory; the domain of executive function includes TMT-B, abstract reasoning, phonemic fluency, clock drawing, and overlapping pictures. The domain of perception includes spontaneous drawing and copy drawing tests. The TMT-B is a well-known instrument for assessing the attentive function, but it also evaluates switching ability and working memory and is thus also considered a measure of executive functions [17]. Given its comprehensive features, we considered ENB-2 as the gold standard to detect MCI.

A further categorization was carried out to identify different subtypes of MCI. Different types of MCI are characterized by the presence of a deficit in more than one single test for each function. For single domain amnestic MCI we considered the impairment in memory and learning functions; multiple domain amnestic MCI was indicated by impaired functions of memory and learning and a low deficiency in other cognitive functions; single domain non-amnestic MCI was indicated by impaired executive or visuospatial functions; for multiple domain non-amnestic MCI we considered the impairment in all cognitive functions with the exclusion of the memory/learning. In conclusion, we defined four dichotomous variables describing the presence/absence of MCI in ENB-2 and we defined MCI as present for ENB-2 = amnestic or non-amnestic impaired domains, and MCI as not present for ENB-2 = amnestic or non-amnestic non impaired domains.

### Statistical analysis

Descriptive statistics are reported as mean ± standard deviation (SD) for continuous variables and as number (percentage of frequency) for discrete variables. The means of the ENB-2 items in the study population were compared with their respective normative sample means using a 2-sided t test. The null hypothesis was that the mean value of the outcome scores was equal to the normative mean for that variable. Between-group comparisons for categorical variables were analyzed by the Chi-square test or by the Fisher exact test when appropriate. The association between dichotomous variables indicating the presence of MCI and clinical parameters—GOLD stage, long-term oxygen therapy (LTOT), demographic (age, sex) and psychosocial variables (depressive and anxiety symptoms)—was assessed by logistic regression. All statistical tests were two-tailed and statistical significance was set at P<0.05. All analyses were carried out using the SAS/STAT statistical package, release 9.2 (SAS Institute Inc., Cary, NC, USA).

## Results

In this multicentre cross-sectional observational study, 98 COPD patients in stage II–IV GOLD, consecutively admitted for inpatient rehabilitation, were screened for inclusion. Among them, 33 patients were excluded for the following reasons: clinical exacerbation during hospitalization (n = 2), relapse into illiteracy (n = 6), visual-perceptive deficits (n = 5), low subjective motivation to undergo the evaluation, or refusal (n = 14), and severe psychiatric diseases (n = 6). The final study population consisted of 65 patients.

Table 1 show the socio-demographic and clinical characteristics of the study population.

**Table 1:** Socio-demographic and clinical characterisitics of the study population (n = 65).

Characteristics	Categories	n (%)
Sex		
	Male	47 (72.3)
	Female	18 (27.7)
Education (years)		
	<5	20 (30.8)
	6–8	31 (47.7)
	9–13	13 (20.0)
	>14	1 (1.5)
Living alone		
	No	51 (78.5)
	Yes	14 (21.5)
Marital Status		
	Married/partner	33 (50.8)
	Widower	16 (24.6)
	Unmarried	10 (15.4)
	Separated/divorced	6 (9.2)
Occupation		
	Retired	61 (93.8)
	Employed	2 (3.1)
	Housekeeper	2 (3.1)
Primary caregiver		
	None	37 (56.9)
	Sibling	13 (20.0)
	Husband/wife/partner	10 (15.4)
	Other	5 (7.7)
Smoker		
	No	3 (4.6)
	Yes	8 (12.3)
	Yes in the past	54 (83.1)
LTOT		
	No	17 (26.2)
	Yes	48 (73.8)
COPD severity (GOLD)		
	I—Mild (FEV1>80%)	0
	II—Moderate (50%≤FEV1<80%)	22 (33.8)
	III—Severe (30≤FEV_1_<50%)	25 (38.5)
	IV—Very severe (FEV1<30%)	18 (27.7)
	**Range**	**M (SD)**
Age, years	53–85	69.9 (7.6)
Duration of illness, months	2–432	117.4 **(**94.4)
6MWT (meters)	80–556	313.9 **(**105.2)
BMI, Kg/m^2^	13.8–59.9	25.9 **(**7.3)
FEV1, L	0.3–2.2	1.1 (0.5)
FEV1%	10–79.7	42.8 **(**17.8)
FVC, L	0.9–4.3	2.4 **(**0.8)
FVC%	42–133	75.1 **(**21.5)
FEV1/FVC	21–69.8	45.1 **(**12.1)

GOLD: Global Initiative for Chronic Obstructive Lung Disease, FEV1: forced expiratory volume in 1 sec, FVC: forced vital capacity, LTOT: long-term oxygen therapy, 6MWT: 6-min walking test; BMI: body mass index.

Table 2 shows the frequency distribution of anxiety and depressive symptoms (HADS, GDS, BDI-II) and of MCI using the various instruments (MMSE vs. MoCA vs. ENB-2).

**Table 2:** Psychological and neuropsychological data of patients (n = 65) from the different screening tests.

Variables	Categories	n (%)
Anxiety symptoms (HADS-A)		
	None	33 (50.8)
	Mild	20 (30.8)
	Moderate	10 (15.4)
	Severe	2 (3.1)
Depressive Symptoms (BDI- II/GDS)		
	None	31 (47.7)
	Mild	14 (21.5)
	Moderate	9 (13.8)
	Severe	11 (16.9)
MCI (MMSE)		
	Yes: 18.3≤x≤23.8	4 (6.2)
	No: >23.8	61 (93.8)
MCI (MoCA–ES*)		
	0 <17.362	3 (4.6)
	1 17.363≤x≤19.500	9 (13.9)
	2 19.501≤x≤21.562	13 (20.0)
	3 21.563≤x≤23.361	12 (18.4)
	4 >23.361	28 (43.1)
MCI (ENB-2)		
	Amnestic single domain	1 (1.5)
	Amnestic multiple domain	17 (26.2)
	Non-amnestic single domain	8 (12.3)
	Non-amnestic multiple domain	7 (10.8)
	No	32 (49.2)

Note

ES* corresponds to a 5-point interval scale divided as follows: 0 = a performance equal to the worst 5% of the normative sample; 4 = scores higher than the median value of the whole sample; 1, 2 and 3 are obtained by dividing into three equal parts the area of the distribution between 0 and 4; 1 could be considered impaired in some clinical condition and settings according to the clinical judgment.

HADS-A: Hospital Anxiety and Depression Scale-Anxiety; BDI-II: Beck Depression Inventory-2^nd^ edition, GDS: Geriatric Depression Scale; MMSE: Mini-Mental State Examination; MCI: mild cognitive impairment; MoCA: Montreal Cognitive Assessment; ES: equivalent scores; ENB-2, Esame Neuropsicologico Breve 2.

The contingency table reporting the association between MCI as assessed by ENB-2 (gold standard) and by MoCA, is reported in Table 3. The association was not significant (p = 0.22), specificity and sensitivity were 0.67 and 0.53 respectively. Furthermore, the positive predictive accuracy value is 0.87 and the negative predictive accuracy value is 0.24.

**Table 3:** Contingency table comparing rate of MCI diagnosis assessed by ENB-2 (gold standard) vs. MoCA.

	ENB-2 MCI Yes	ENB-2 MCI No
	n (%)	n (%)
**MoCA MCI Yes**	28 (43.1%) True positive	4 (6.1%) False positive
**MoCA MCI No**	25 (38.5%) False negative	8 (12.3%) True negative

Note

Chi-square test (p = 0.22), specificity (0.67) and sensitivity (0.53), positive predictive accuracy (0.87) and negative predictive accuracy (0.24).

MCI: mild cognitive impairment; MoCA: Montreal Cognitive Assessment; ENB-2, Esame Neuropsicologico Breve 2.

Table 4 reports the comparison between ENB-2 scores of COPD patients vs. normative data. In addition, we calculated the frequency distribution (percentage) of impaired scores for each ENB-2 test. Patients performed significantly worse compared to the Italian normative sample in the following domains: anamnestic (digit span) and non-anamnestic (TMT-B, overlapping pictures and copy drawing tests). Regarding the TMT-A test, COPD patients were more likely to have a better performance in comparison to the normative sample (55.2±31.6 vs. 81.53±33.9, p = 0.0001). In the TMT-A test, the frequency (percentage) of patients with deficit scores under the normative data was not high (n = 4, 6.2%).

**Table 4:** Scores on ENB-2 neuropsychological tests: comparison with normative data, and number (% frequency) of impaired scores.

Cognitive Domain	ENB-2 tests	COPD patients (n = 65)	Normative data (n = 372)	2-sided T test	Impaired scores
		M (DS) score	M (DS) score	p-value	n (%)
Anamnestic					
	Digit span	5.11 (0.9)	5.52 (1.0)	0.0004	5 (7.7)
	Episodic memory: IR	12.11 (4.8)	11.48 (3.9)	ns	7 (10.8)
	Episodic memory: DR	15.26 (4.8)	14.56 (4.6)	ns	4 (6.2)
	Interference memory 10”	5.62 (2.5)	5.84 (2.2)	ns	15 (23.1)
	Interference memory 30”	5.23 (2.6)	5.24 (2.1)	ns	11 (16.9)
Non-anamnestic Executive Function					
	TMT-A	55.2 (31.6)	81.53 (33.9)	0.0001	4 (6.2)
	TMT-B	176.31 (99.5)	135.93 (58.0)	0.004	20 (30.8)
	Abstract verbal reasoning	3.95 (1.9)	4.41 (4.6)	ns	18 (27.7)
	Phonemic fluency	10.88 (3.8)	10.72 (3.9)	ns	6 (9.2)
Non-anamnestic Visuospatial/praxis skills					
	Clock drawing	8.78 (1.8)	8.34 (1.8)	ns	3 (4.6)
	Overlapping pictures	26.03 (8.9)	28.75 (8.2)	0.018	15 (23.1)
	Spontaneous drawing	1.75 (0.6)	1.88 (0.4)	ns	6 (9.2)
	Copy drawing	1.37 (0.6)	1.61 (0.5)	0.002	15 (23.1)
	Ideative/ideomotor praxis	5.66 (0.7)	5.81 (0.4)	ns	9 (13.8)

ENB-2, Esame Neuropsicologico Breve 2; IR: immediate recall; DR: delayed recall: TMT-A: trail making test A; TMT-B: trail making test B.

At logistic regression analysis, except for copy drawing, no significant association was found between candidate predictors and impaired performance assessed by the neuropsychological tests (digit span, TMT-A, TMT-B, abstract verbal reasoning, and overlapping pictures). Among the set of variables considered as potential predictors (gender, age, COPD GOLD-severity, LTOT, anxiety and depressive symptoms), only COPD severity (p = 0.012, odds ratio, OR, 4.4 [95% confidence interval, CI: 1.4–14.0]), and anxiety symptoms (p = 0.026, OR 4.6 [95% CI: 1.2–17.7]) were identified as significant and independent predictors of the deficit in the copy drawing test. Hence, for a unit increase in GOLD stage or in anxiety symptoms there was respectively a 3.4-fold and 3.6-fold increase in the odds of a deficit in the copy drawing test. The association with gender (p = 0.057, males at higher risk) and with depressive symptoms (p = 0.057, inverse association) was only borderline significant.

## Discussion

Our study provides an analysis, more in-depth with respect to previous publications, of the neuropsychological impairments in COPD patients. It assessed MCI by means of both neuropsychological screening tests and a complete neuropsychological battery, and compared COPD neuropsychological data with those of a normative sample. In addition, it analysed the relationship between cognitive performance, and clinical and psychological variables.

Concerning the moderate-to-severe levels of anxiety and depressive symptoms found, our data are in line with the literature [32, 18] and the prevalence of symptoms is, respectively, 18.5% and 30.7%. It is thus important to screen psychological distress, given the significance of anxiety and depressive symptoms in this population and the fact that there is a significant overlap with symptoms of COPD (e.g. sleep disorders, fatigue, and loss of independence in daily life activities) [19, 32]. The use of self-reported questionnaires, instead of well-structured but time-consuming interviews, can give an immediate first overview of these multisystem symptoms and identify patients to be subsequently examined in depth through psychological or psychiatric counselling in order to program more tailored interventions.

Regarding the cognitive aspects, we found different percentages of MCI prevalence in the COPD sample depending on the type of neuropsychological test used (Table 2): 6.2% (MMSE), 18.5% (MoCA) and 50.8% (ENB-2). As is well known, MMSE is not the best suited instrument for detecting MCI; MoCA is better able to identify MCI than MMSE thanks to its executive tests, which make it possible to identify non-amnestic MCI [14, 15], also in COPD patients [3, 4, 12]. ENB-2 resulted a gold standard to detect MCI (Table 3). Furthermore, through the ENB-2 we were able to identify not only a high percentage of non-anamnestic single or multiple domain MCI (23.1%), but also a high percentage of amnestic single or multiple domain MCI (27.7%). Our data are in line with Singh et al. who found in elderly COPD patients (70–89 years) a higher percentage of both anamnestic and non-anamnestic MCI compared to patients without COPD [9].

Our MoCA results warrant a specific comment. It appears, considering the international literature, that the prevalence of MCI in our sample is lower compared to non-Italian cohorts [4, 12]. This could be due to the use in each country of different cut-off scores to define MCI. This is an extremely important issue that points to the need for further dedicated studies in order to develop standard criteria valid for all countries (15). Furthermore, the comparison between ENB-2 impaired scores and the MoCA ES data highlights the higher sensitivity of ENB-2 to detect MCI and warns of the risk of false negatives (n = 25) with using only the MoCA test.

Examining more deeply the cognitive functioning results from the ENB-2 (Table 4), COPD patients performed significantly worse than the Italian normative sample in the following domains: anamnestic (digit span test) and non-anamnestic (TMT-B, overlapping pictures and copy drawing test). This confirms the presence of impairment in both anamnestic and non-anamnestic functioning in these patients and a significant decline across different cognitive functions. In fact, we found that only 16.9% of our sample had no impaired scores. On the contrary, 83.1% of COPD patients in our sample had an impaired score on at least one test: 33.9% on only 1, 36.9% on 2–4 tests, and 12.3% on 5–8 tests, indicating poor performance across a broad range of cognitive domains.

In a recent study, Singh et al. found that the association between COPD and MCI was significant (p = .0003) even after adjusting for age, education, gender, depression, cardiovascular comorbidities and other covariates. This strengthens the evidence that the association existing between COPD and MCI is independent of comorbidities and probably associated with the chronic inflammatory process, which may have a role in the cognitive impairment. In addition, in this longitudinal study, there was a dose-response relationship between duration of over 5 years from baseline and risk of MCI in COPD patients [9]. It is not simple to determine the risk of developing more severe cognitive and functional impairments in patients with COPD and MCI. Evidence suggests that, in patients with severe COPD, cognitive deficits worsen over time, and so these patients are more likely to develop Alzheimer’s disease or vascular dementia. Cognitive decline in COPD patients is associated with high mortality and disability [3, 33–35].

Furthermore, our findings indicate that increasing COPD severity (i.e. progressing to the next GOLD stage) is associated with a 4.4-fold increased risk of impaired performance in the copy drawing test, and passing from one level of anxiety to the next is associated with a 4.6-fold increased risk of impaired performance. Adding this result to the documented evidence of the copy drawing test as a risk factor for mortality in COPD patients [34], we support Antonelli-Incalzi et al.’s recommendation that this brief neuropsychological test be administered as part of the routine COPD patient assessment. Also in the recent literature it was suggested, based on a significant correlation of anxiety, depression and COPD severity with cognitive impairment, to assess depression and anxiety [24].

In conclusion, in the light of evidence from the literature and our own findings, it may be useful to routinely screen COPD patients for the presence of both psychological and neuropsychological comorbidities. The choice of an appropriate cognitive screening tool should be guided by: the clinical utility (easy to use and fast to administer), good psychometric properties such as high sensitivity (correct identification of true positives), good specificity (correct identification of true negatives), and the presence of normative data. Therefore, for the cognitive domain, we recommend adding to the MoCA test some other neuropsychological tests such as the copy drawing test (given its significant relationship with disease severity and anxiety), the TMT-B (due to its capacity to detect switching ability) and the digit span test (for a simple and fast survey of working memory). This detailed assessment could be effective in detecting different types of MCI. In addition, it could provide effective tools for everyday patient management, given the increasing importance of personalised activities in pulmonary rehabilitation and the need to motivate COPD patients to achieve the goals they perceive as the most relevant for their daily life, regardless of the disease severity and the presence of depression or anxiety [36, 37].

Our study has some strengths and limitations. The strong points are the use of a broad neuropsychological battery with normative data and the analysis of the relationship between clinical, psychological and neuropsychological variables. The main limitations are the lack of generalizability of our data to the COPD population as a whole due to the nature of our sample (i.e. inpatients who voluntarily attended rehabilitation departments), and the small sample size (particularly in view of the high number of variables examined).

## Conclusion

The use of adequate screening tools for mood, cognitive and anxiety multisystem symptoms in COPD patients can help detect the presence of these important comorbidities, which often remain hidden, overlapped and underestimated, and ensure a more individually-tailored interdisciplinary rehabilitation of COPD patients.

## Supporting information

S1 Dataset (XLSX)
